# Comprehensive analysis of the multifaceted role of ITGAV in digestive system cancer progression and immune infiltration

**DOI:** 10.3389/fimmu.2025.1480771

**Published:** 2025-02-13

**Authors:** Xinyue Shi, Jingyu Zang, Qi Gu, Mengmeng Zhang, Handi Sun, Lijun Yang, Jiahui Cheng, Ruonan Wang, Han Mao, Aitong Xu, Xin Wang, Yu Xiao, Jialing Cai, Fang Han, Depeng Yang, Yu Li, Huan Nie

**Affiliations:** ^1^ School of Life Science and Technology, Faculty of Life Sciences and Medicine, Harbin Institute of Technology, Harbin, Heilongjiang, China; ^2^ Department of Toxicology, College of Public Health, Harbin Medical University, Harbin, Heilongjiang, China

**Keywords:** ITGAV, diagnosis, migration, immunology, digestive system cancers

## Abstract

**Background:**

Digestive system cancers are among the most common malignancies, exhibiting consistently high incidence and mortality rates, yet effective detection and treatment targets remain limited. Integrin αv (ITGAV, CD51) is a significant member of the integrin family, widely recognized for its role in mediating interactions between cells and the extracellular matrix, as well as intracellular signaling. In recent years, ITGAV has been found to have significantly elevated expression in multiple tumors, such as prostate cancer, breast cancer, and osteosarcoma, and was considered to be a key component in various stages of tumor progression. However, no systematic digestive system cancer analysis has been conducted to explore its function in prognosis, diagnosis, and immunology.

**Methods:**

Transcriptome sequencing and clinical data of samples were obtained from The Cancer Genome Atlas (TCGA), Genotype-Tissue Expression Project (GTEx), Human Protein Atlas (HPA), cBioPortal, TIMER and TISIDB databases. Bioinformatics methods were employed to investigate the potential oncogenicity of ITGAV, focusing specifically on the analysis of its prognosis, diagnostic value, and immune infiltration level of ITGAV in digestive system cancers. In addition, GO, KEGG, and PPI network analysis revealed the biological functions and related signaling pathways related to ITGAV. Finally, the role of ITGAV in regulating cancer progression was experimentally verified using hepatocellular carcinoma and pancreatic cancer as examples.

**Results:**

We found that ITGAV was highly expressed in multiple digestive system cancers. In addition, high expression of ITGAV was closely associated with poor prognosis and showed potential for early diagnosis. Enrichment of pathways related to extracellular matrix organizing processes and tumor migratory movements was identified. *In vitro*, results showed that the knockdown of ITGAV significantly inhibited the migratory movement ability of hepatocellular carcinoma and pancreatic cancer cells, while its overexpression significantly promoted the migration of the above cells. Finally, immunoassays showed a significant correlation between ITGAV expression and the infiltration level of various immune cells, further clarifying the critical role of ITGAV in the tumor immune microenvironment.

**Conclusion:**

Our results elucidated the importance of ITGAV in the prognostic assessment, early diagnosis, and targeted immunotherapy of digestive system cancers, and revealed its multifaceted role in regulating cancer progression.

## 1 Introduction

Digestive system cancers account for more than one-fourth of all tumor cases and one-third of cancer-related deaths globally, with a large number of cases and a wide range of locations, causing tremendous suffering and economic burden to patients, families, and healthcare systems (1, 2). Among digestive system cancers, stomach, pancreatic, colon, liver, and esophageal cancers are the main types of cancer (3). The International Agency for Research on Cancer (IARC) of the World Health Organization has reported a concerning incidence and mortality growth from digestive system cancers worldwide (4, 5). Despite advances in the treatment of digestive system cancers, such as the widespread removal of tumors and the use of new drugs in chemotherapy, the long-term survival rate of patients with this type of cancer is still much lower than that of patients with other major cancer sites (6). Therefore, there is an urgent need to discover a new, reliable prognostic marker and an effective therapeutic target for tumors to improve clinical outcomes and reduce case burden.

Integrin is a cell adhesion molecule comprising two subunits, α and β (7). As a member of the cell membrane protein receptors, integrin mediates cell-to-cell interactions and possesses bidirectional signaling capabilities across the cell membrane (8). At the same time, it has a very significant role in regulating the biological functions of cells and influencing tumor proliferation, apoptosis, invasion, cell transformation, angiogenesis, and drug resistance (9–11). Integrin αv (ITGAV, CD51) is one of the crucial members of the integrin family and is regarded as a key component in multiple stages of tumor progression (12). In recent years, aberrant expression of ITGAV has been clearly identified in various tumor studies, suggesting that ITGAV is closely associated with tumor development and metastasis. For example, large extracellular vesicles (EVs) overexpressing ITGAV promoted prostate cancer (PCa) adhesion and invasion via AKT activation (13). In glioblastoma, the mitochondria-derived peptide humanin enhanced its invasion and migration through intratumoral activation of the ITGAV-TGFβ signaling axis (14). It had been reported that ITGAV was a promising therapeutic target for esophageal squamous cell carcinoma (ESCC), as indomethacin can inhibit ESCC growth by binding to ITGAV, promoting SYVN1-mediated ubiquitination of ITGAV, and enhancing cytotoxic CD8^+^ T cell responses (15). In addition, high expression of ITGAV significantly influenced the level of immune cell infiltration in lung adenocarcinoma and Triple-Negative breast cancer and was associated with poor prognosis in head and neck squamous cell carcinoma (HNSCC) and osteosarcoma (16–19). Furthermore, the major downstream kinase of ITGAV had been shown to be of diagnostic value as an independent prognostic factor in patients with liver cancer after surgery, predicting the survival outcome of patients. Although these findings suggested the potential of ITGAV as a therapeutic target and prognostic marker, its potential function, prognostic value, and immune characteristics in digestive system cancers remain unclear (20). Here, we comprehensively investigated the different expression levels of ITGAV in digestive system cancer samples and normal samples by databases. Meanwhile, the diagnostic and prognostic value of ITGAV in digestive system cancers was evaluated, and the ITGAV genomic variants were analyzed. Subsequently, the function of ITGAV in digestive system cancers was described based on bioinformatics analysis, and these findings were validated through *in vitro* experiments. Finally, its potential relationship with immune infiltration, immune checkpoints, TMB, MSI, immunomodulators, chemokines, and chemokine receptors was explored. Our study reveals the potential of ITGAV as a diagnostic and prognostic biomarker, while also demonstrating its key regulatory role in immunotherapy and tumor metastasis, providing a promising novel target for digestive system cancers treatment options.

## 2 Materials and methods

### 2.1 Analysis based on computational procedure

#### 2.1.1 Data acquisition and gene expression analysis

Firstly, the mRNA expression profiles of ITGAV in tumor samples, corresponding adjacent normal samples, and healthy samples, along with relevant clinical data such as pathological stage and TNM stage, were obtained from the Cancer Genome Atlas (TCGA) dataset (https://cancergenome.nih.gov) and the Genotype-Tissue Expression (GTEx) dataset (https://commonfund.nih.gov/GTEx). These data were systematically sorted out and standardized for subsequent visualization analysis. Subsequently, immunohistochemical (IHC) staining images of ITGAV in different tumor tissues and their corresponding normal tissues were downloaded from the Human Protein Atlas (HPA) dataset (https://www.proteinatlas.org/) to assess the differences in ITGAV expression at the protein level. Finally, we used the TNMplot (https://tnmplot.com/analysis/) online server and its Kruskal-Wallis test to evaluate the relationship between ITGAV expression in tumor, normal, and metastatic tissues.

#### 2.1.2 Survival prognosis and roc diagnosis analysis

We also downloaded survival data from TCGA for five cancer samples: gastric adenocarcinoma (STAD), pancreatic adenocarcinoma (PAAD), hepatocellular carcinoma (LIHC), esophageal carcinoma (ESCA), and colorectal carcinoma (COAD), and divided the patients into a high-expression group (High) and a low-expression group (Low). Overall survival (OS), disease-specific survival (DSS), and progression-free interval (PFI) were considered as indicators to explore the relevance between ITGAV expression and patient prognosis. Survival analysis was performed using the “survival” package for Kaplan - Meier (KM) analysis and Cox regression tests, and the results were visualized using the “survminer” package as well as the “ggplot2” package. To assess the diagnostic accuracy of ITGAV, statistical analyses based on sensitivity and specificity were performed using the “pROC” R package, and the “ggplot2” R package was used to create subject work characteristics (ROC) curves. ROC curves of ITGAV with an Area Under the Curve (AUC) of more than 0.7 were regarded as high diagnostic values in different types of human cancers.

#### 2.1.3 Gene alteration analysis

The cBioPortal database (http://www.cbioportal.org/) was used to analyze the gene alternations of ITGAV in TCGA pan-cancer datasets. The “Cancer Types Summary” module shows the target genes’ mutation frequency and type results in a bar chart format. Furthermore, “OncoPrint” shows the mutations, copy number, and expression of the target genes in all samples in the form of a heat map. Finally, the “Mutations” module was utilized to search for mutation sites and protein post-translational modification sites.

#### 2.1.4 Functional enrichment and protein-protein interaction network analysis

The samples in TCGA were categorized into ITGAV high and low expression groups based on the median ITGAV mRNA expression level. Differentially expressed genes (DEGs) between ITGAV high and low expression groups were identified using the R package “Limma” with screening criteria of | log2FC | > 1.5 and adj *p* < 0.05. Gene ontology (GO) and KEGG pathway analysis of DEGs in the ITGAV high and low expression groups was performed using the R package “ClusterProfiler”. The results are presented as a chord diagram via the “ggplot2”.

To construct a network of ITGAV-interacting proteins, we accessed the STRING database (21). By setting the interaction score to “high confidence”, we identified the top 50 interacting proteins, which were subsequently visualized as a protein-protein interaction (PPI) network using Cytoscape (version 3.9.1). These genes were enriched by GO and KEGG pathway analysis. Both GO annotation enrichment analysis and KEGG pathway enrichment analysis were considered statistically significant at *P* < 0.05.

#### 2.1.5 Co-expressed genes correlation analysis

Then, we accessed the cBioPortal database to identify data co-expressed with ITGAV using Spearman’s correlation coefficient |R| >0.5, *P*-value <0.05 as the screening index. Subsequently, the heatmap of ITGAV-related and interacting genes was generated using the “Gene Corr” module of Timer2.0 (http://timer.comp-genomics.org/timer/), and the scatterplot of the correlation was further plotted using the Gene Expression Profiling Interactive Analysis (GEPIA) database (http://gepia2.cancer-pku.cn/).

#### 2.1.6 Immune characteristics analysis

The ESTIMATE algorithm was used to assess the relationship between ITGAV expression and Immune, Stromal, and ESTIMATE scores in STAD, PAAD, LIHC, ESCA, and COAD samples. More importantly, the infiltration levels of 24 immune cells in tumor samples were explored using the “GSVA” package and the “ssGSEA” algorithm, and Spearman’s correlation coefficients were used to calculate the correlation between the levels of immune cell infiltration and the levels of ITGAV expression. The TIMER2.0 database was also used to explore the correlation between ITGAV expression and the infiltration levels of macrophages, Th17 cells, and CAFs infiltration levels. In addition, the relationship between ITGAV and immune checkpoints, tumor mutation burden (TMB), microsatellite instability (MSI), and homologous recombination defects (HRD) was also evaluated using the SangerBox web server. Finally, we explored the correlation of ITGAV expression with molecular subtypes, immune subtypes, and immune modulators from the TISIDB database (http://cis.hku.hk/TISIDB/index.php), which integrates multiple data types to assess tumor-immune system interactions (22).

### 2.2 Analysis based on laboratory procedure

#### 2.2.1 Clinical samples, cell lines, and cell culture

All hepatocellular carcinoma tumor tissue samples used in this research were obtained from Harbin Medical University and had patient informed consent. This study has been approved by the Medical Ethics Committee of Harbin Institute of Technology (Approval Number: HIT-2022008).

The human hepatocarcinoma cell lines (MHCC97H, HepG2, PLC/PRF/5, HuH-7) and the human pancreatic cancer cell lines (PANC-1, MIA PaCa-2, BxPC-3, AsPC-1) were obtained from Stem Cell Bank, Chinese Academy of Sciences. They were routinely tested to exclude mycoplasma contamination and authenticated by Genetic Testing Biotechnology (Suzhou, China). All cells were cultured in Dulbecco’s modified Eagle’s medium (DMEM) or RPMI-1640 medium, supplemented with 10% fetal bovine serum (FBS) and 1% penicillin/streptomycin and cultured at 37°C in a humidified incubator with 5% CO_2_.

#### 2.2.2 The siRNA, plasmid construction, and cell transfection

The siRNA specifically targeting ITGAV was synthesized by General Biol (Anhui). ITGAV plasmid was designed and constructed using human ITGAV and cloned into the plvsin-EGFP vector. A plvsin-EGFP empty vector was used as a negative control. All cells were transfected with Lipofectamine 2000 (Invitrogen) according to the manufacturer’s protocol. The cells were collected for subsequent experiments following 48 h of transfection.

#### 2.2.3 Quantitative reverse transcription-polymerase chain reaction

The cDNA was synthesized using the Prime-script™ RT reagent kit with gDNA Eraser (TAKARA) following total RNA purification with TRIzol reagent according to the manufacturer’s instructions. Quantitative reverse-transcriptase PCR experiments were conducted using the reaction mix of SYBR Green. The expression level was determined using the 2^-ΔΔCt^ method and normalized to the housekeeping gene GAPDH.

#### 2.2.4 Western blot

All cells were lysed by using RIPA lysis buffer containing protease inhibitors to release the proteins. Subsequently, the supernatant was carefully collected after centrifuging at 12000 g for 15 min at 4°C and then boiled in a loading buffer to make the protein denaturation. The protein samples were separated by 10% -PAGE and were transferred onto polyvinylidene difluoride (PVDF) membranes. After being blocked with 5% nonfat milk for 1 h, the membranes were incubated with primary antibodies overnight at 4°C and then bound to the secondary antibodies at room temperature for 1 h. The automatic chemiluminescence analysis system (Tanon 5200) was used to visualize the immunoreactive substrates. The Prestained Protein Marker was purchased from Thermo Fisher. The Image J software analyzed the images to determine the relative protein expression level.

#### 2.2.5 Wound healing assay

For the wound healing assay, all cells were incubated in 24-well plates. The middle of these wells was scratched at approximately 0.4-0.5 mm width when they had been spread almost evenly over the bottom of these wells to form a monolayer. After that, these cells were treated with a medium containing 2% FBS. The images were captured at the beginning and every 24 h and were analyzed to quantify the wound closure by measuring the width of the scratch at regular intervals and comparing them to the initial width.

#### 2.2.6 Transwell chamber-based migration assay

For the transwell chamber-based migration assay, these cells were resuspended in a serum-free medium and inoculated in the upper compartment of the chamber. Subsequently, a complete medium was added to the lower chamber. The cells were fixed and stained with 0.3% crystal violet at room temperature following a 48 h incubation period at 37°C. Images were photographed using an inverted microscope and analyzed by the Image J software.

#### 2.2.7 Tissue immunofluorescence

Frozen tissues from HCC patients were cut into 10 μm thick sections. The sections were left at room temperature for 30 min and then fixed with 4% paraformaldehyde (PFA) for 15 min. After sealing with 5% BSA, the sections were incubated overnight with the appropriate primary antibody and fluorescent dye-coupled secondary antibody for 1 h. After incubation with DAPI for 10 min, images were acquired using a laser confocal microscope.

#### 2.2.8 Statistical analysis

The experimental data were presented as the mean ± SEM. The statistical analysis of the differences between the two groups was performed using a two-tailed Student’s t-test, which was analyzed using GraphPad Prism 8. The significance thresholds were defined as statistically significant (* *P* < 0.05), highly significant (** *P* < 0.01), or extremely significant (*** *P* < 0.001). ns, not statistically significant.

## 3 Results

### 3.1 Expression and mutation analysis of ITGAV in digestive system cancers

**Figure 1:**
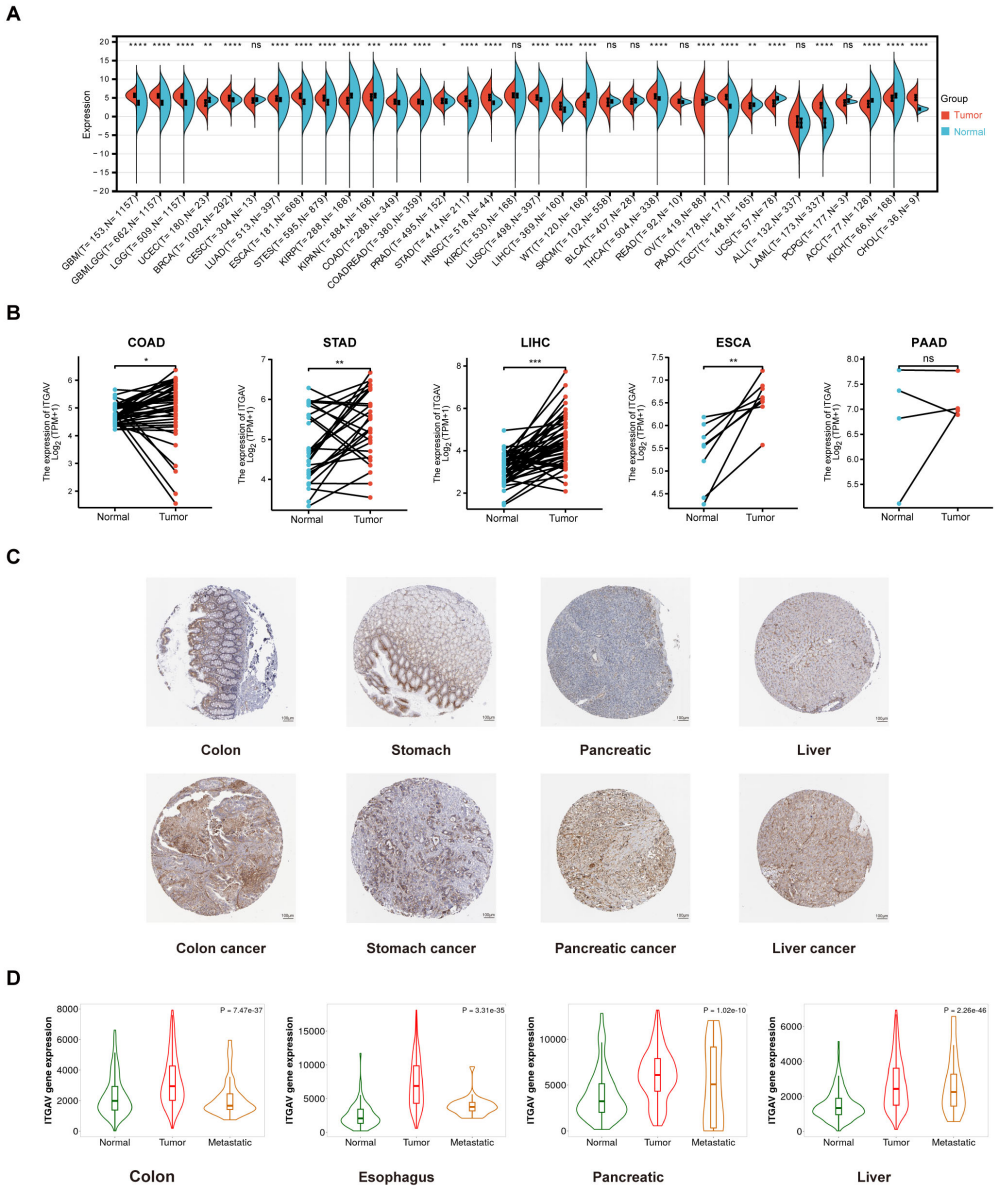
ITGAV up-regulation in multiple digestive system cancers. **(A)** Expression of ITGAV mRNA in pan-cancers. **(B)** Comparison of ITGAV mRNA expression between tumor and paired normal samples. **(C)** The protein expression of ITGAV in immunohistochemical images of normal (up) and tumor (down) groups. **(D)** Correlation between ITGAV mRNA expression and tissue type (Normal/Tumor/Metastatic). *p < 0.05, **p < 0.01, ***p < 0.001, ****p < 0.0001. ns, not statistically significant. COAD, colorectal adenocarcinoma; STAD, stomach adenocarcinoma; LIHC, hepatocellular carcinoma; ESCA, esophageal adenocarcinoma; PAAD, pancreatic adenocarcinoma.

To investigate the expression of ITGAV in multiple digestive system cancers, we first examined the expression of ITGAV in tumor samples and normal samples using TCGA as well as GTEx databases, which showed that ITGAV expression was elevated in the majority of the tumors (18 of 34). It is worth noting that, among these types of tumors, we found that ITGAV expression was significantly higher in multiple digestive system cancer tissues than in normal tissues, including COAD, ESCA, LIHC, PAAD, and STAD (*P* < 0.001) (**Figure 1A**). Whereafter, we also made a comparison between tumor and paired normal samples on ITGAV expression levels in these digestive system cancers, based on TCGA data (**Figure 1B**). The results showed that ITGAV was significantly highly expressed in COAD, ESCA, LIHC, and STAD. However, there was no significant difference observed in PAAD, likely due to the limited sample size (n=4). These findings were subsequently confirmed by analyzing the ITGAV mRNA expression levels in these tumors and their respective normal tissues, sourced from the GEPIA database (**Supplementary Figures S1A–E**). Following that, immunohistochemical images sourced from the HPA database were utilized to assess the protein-level expression of ITGAV. The analysis revealed that the protein expression of ITGAV in COAD, LIHC, PAAD, and STAD was significantly elevated compared to that in normal tissues (**Figure 1C**). In addition, the correlation between ITGAV mRNA expression levels and metastases was examined using the TNMplot online server. Upon comparing normal and tumor tissues, ITGAV expression was markedly increased in the colon, pancreas, liver, and esophagus. This elevated expression pattern was consistent between metastatic and cancerous tissues, as depicted in **Figure 1D**.

To further reveal the relationship between ITGAV expression levels in clinical samples and the malignant progression of digestive system cancers, we used an online analysis tool to group the four clinical samples of digestive system cancers, STAD, COAD, LIHC, and ESCA, in the TCGA database according to T-stage, N-stage, M-stage, and pathological stage. Subsequently, we analyzed the expression of ITGAV. We discovered a consistent elevation in ITGAV expression levels with increasing tumor malignancy across T-stage, N-stage, M-stage, and pathological stage categories (**Supplementary Figures S2A–D**). These results indicate that ITGAV expression was elevated in multiple digestive system cancers, such as COAD, STAD, PAAD, LIHC, and ESCA, suggesting that ITGAV might serve as a crucial factor in cancer diagnosis.

**Figure 2:**
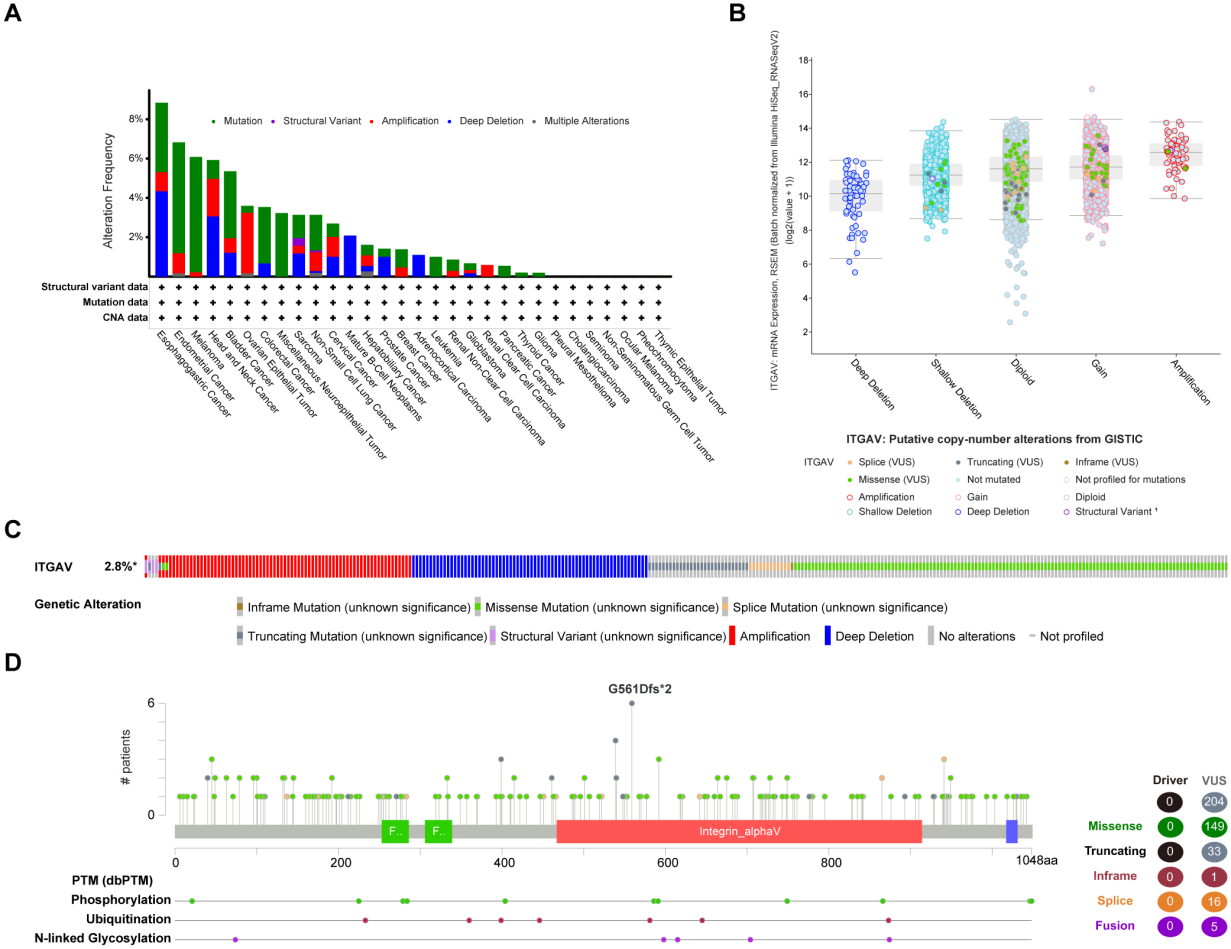
Mutation assessment for ITGAV using the cBioPortal tool. **(A)** ITGAV mutation types and frequency. **(B)** ITGAV copy number alterations (CNA) types. **(C)** OncoPrint visual summary of ITGAV structural variant, mutations, and copy-number alterations. **(D)** The mutation number, and sites of the ITGAV genetic alterations.

Next, we analyzed mutations in the ITGAV gene in all tumor tissues through the cBioPortal platform (**Figure 2**). Patients with ESCA and LIHC showed similar proportions of mutations and deep deletions, while patients with COAD and PAAD primarily displayed genetic alterations in the form of mutations. Moreover, patients with gastric adenocarcinoma had the highest frequency of ITGAV gene alterations among the deep deletion types (**Figure 2A**). The most common putative copy number alterations (CNA) in ITGAV are amplification, gain, diploidy, shallow deletion, and deep deletion (**Figure 2B**). Most cancers investigated exhibit genetic alterations in the form of mutations, amplifications, or deep deletions as the predominant form (**Figure 2C**). Subsequently, we investigated the types, loci, and number of instances of ITGAV gene modifications and found a total of 204 mutation sites, with missense mutations leading the list (149 samples), 33 truncation mutations, 16 splicing mutations, 5 fusion mutations, and the G561DFS*2 type as the most common mutation site (**Figure 2D**). Furthermore, ITGAV was observed to be regulated by post-translational modifications, including phosphorylation, ubiquitination, and *N*-linked glycosyla. These findings are significant for revealing the molecular mechanism of ITGAV in digestive system cancers occurrence and development.

### 3.2 Prognostic assessment and diagnostic value of ITGAV in digestive system cancers

**Figure 3:**
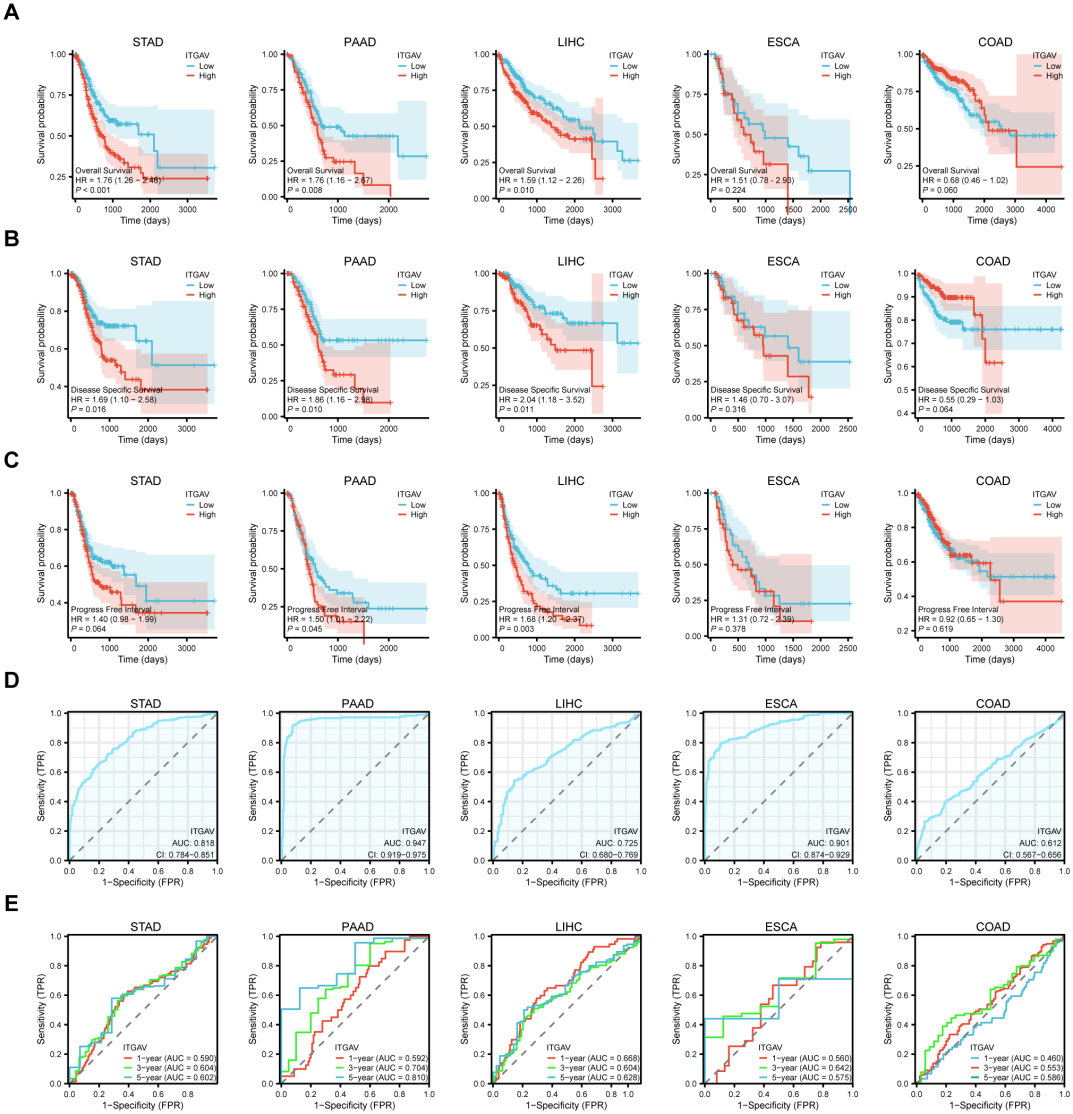
Significant correlation between ITGAV gene expression and prognosis and diagnosis. **(A)** Association between ITGAV expression and overall survival (OS). **(B)** Association between ITGAV expression levels and disease-specific survival (DSS). **(C)** Association between ITGAV expression levels and progression-free interval (PFI). **(D)** Receiver Operator Characteristic (ROC) curve of ITGAV in STAD, PAAD, LIHC, ESCA and COAD. **(E)** ROC curves of ITGAV for predicting 1/3/5-year survival.

To explore the correlation between ITGAV expression levels and prognosis, we performed survival association analyses for a selection of the digestive system cancers mentioned above, with a focus on Overall Survival (OS), Disease-Specific Survival (DSS), and Progression-Free Interval (PFI). The Kaplan-Meier survival curve analysis results demonstrated that ITGAV upregulation was associated with overall survival in STAD (*P* < 0.001), PAAD (*P* < 0.01), and LIHC (*P* < 0.05) (**Figure 3A**). Disease-specific survival analysis yielded similar results (**Figure 3B**). In terms of progression-free survival, LIHC (*P* < 0.01) as well as PAAD (*P* < 0.05) patients with ITGAV overexpression also had poor survival (**Figure 3C**). The findings indicated that ITGAV expression can accurately predict the prognosis of patients with four digestive system cancers: STAD, PAAD, ESCA, and LIHC.

Subsequently, The ROC curves were utilized to assess the performance of the ITGAV gene signature for diagnostic accuracy. As shown in **Figure 3D**, ITGAV has a good diagnostic value in digestive system cancers, including LIHC (AUC = 0.725), STAD (AUC = 0.818), COAD (AUC = 0.612), ESCA (AUC = 0.901) and PAAD (AUC = 0.947),COAD (AUC = 0.612). At the same time, we established time-dependent survival Receiver Operating Characteristic (ROC) curves using ITGAV expression in our samples to forecast 1-year, 3-year, and 5-year survival rates, which also showed that the AUC values were all greater than 0.5 (**Figure 3E**). These results indicate a potential association between ITGAV and the malignant progression of digestive system cancers and may have important clinical value in the early diagnosis of these tumors.

### 3.3 Enrichment analysis of ITGAV in digestive system cancers

According to the above findings, ITGAV exhibits a significant correlation with the survival of patients with digestive system cancers. Therefore, it is crucial to explore the function of ITGAV in multiple digestive system cancers.

**Figure 4:**
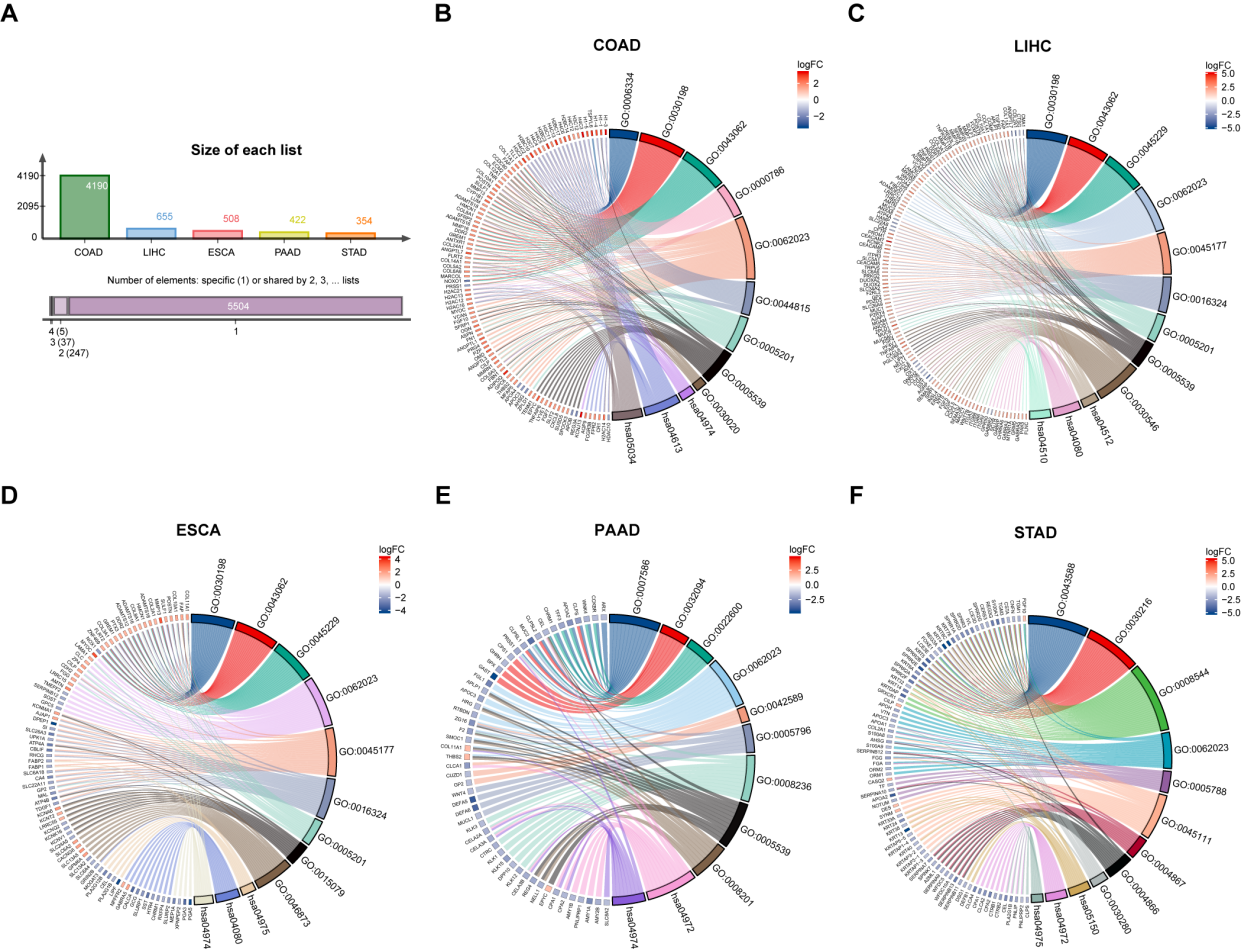
GO and KEGG analysis of DEGs in digestive system cancers by ITGAV. **(A)** ITGAV differentially expressed genes in digestive system cancers. GO and KEGG analysis of ITGAV in **(B)** COAD. **(C)** LIHC. **(D)** ESCA. **(E)** PAAD. **(F)** STAD.

Firstly, | log_2_FC | > 1.5 and adj *p* < 0.05 were used as criteria to obtain ITGAV expression profile data. A total of 4190, 655, 508, 422, and 354 genes differentially expressed with ITGAV were identified in COAD, LIHC, ESCA, PAAD, and STAD, respectively (**Figure 4A**). Next, The GO and KEGG pathways were analyzed for these genes (**Table 1**). The results that showed the key entries in the categories of biological process (BP), cellular component (CC), molecular function (MF), and KEGG pathways across the five types of tumors investigated in our study. As the results of the biological process study suggest, ITGAV-associated DEGs may be closely related to nucleosome assembly, digestive system processes, extracellular matrix, or structural organization processes (**Figures 4B–F**). Regarding cellular components, most genes were mainly distributed in integrated components such as collagen-containing extracellular matrix, apical plasma membrane, and luminal side of the endoplasmic reticulum (**Figures 4B–F**). When assessed for molecular function, these differentially expressed genes show binding activity to structural components of the extracellular matrix, cell adhesion molecules, and metal ion transmembrane transporter (**Figures 4B–F**). Meanwhile, KEGG pathway analysis showed that ITGAV was associated with ECM-receptor interaction, focal adhesion, and protein digestion and absorption (**Figures 4B–F**).

**Table 1:** GO and KEGG enrichment pathways summary.

Category	ID	Description
BP	GO:0006334	nucleosome assembly
	GO:0030198	extracellular matrix organization
	GO:0043062	extracellular structure organization
	GO:0045229	external encapsulating structure organization
	GO:0007586	digestion
	GO:0032094	response to food
	GO:0022600	digestive system process
	GO:0043588	skin development
	GO:0030216	keratinocyte differentiation
	GO:0008544	epidermis development
CC	GO:0000786	nucleosome
	GO:0062023	collagen-containing extracellular matrix
	GO:0044815	DNA packaging complex
	GO:0045177	apical part of cell
	GO:0016324	apical plasma membrane
	GO:0042589	zymogen granule membrane
	GO:0005796	Golgi lumen
	GO:0005788	endoplasmic reticulum lumen
	GO:0045111	intermediate filament cytoskeleton
MF	GO:0005201	extracellular matrix structural constituent
	GO:0005539	glycosaminoglycan binding
	GO:0030020	extracellular matrix structural constituent conferring tensile strength
	GO:0015079	potassium ion transmembrane transporter activity
	GO:0046873	metal ion transmembrane transporter activity
	GO:0030546	signaling receptor activator activity
	GO:0008236	serine-type peptidase activity
	GO:0008201	heparin binding
	GO:0004867	serine-type endopeptidase inhibitor activity
	GO:0004866	endopeptidase inhibitor activity
	GO:0030280	structural constituent of skin epidermis
KEGG	hsa04974	Protein digestion and absorption
	hsa04613	Neutrophil extracellular trap formation
	hsa05034	Alcoholism
	hsa04975	Fat digestion and absorption
	hsa04080	Neuroactive ligand-receptor interaction
	hsa04512	ECM-receptor interaction
	hsa04510	Focal adhesion
	hsa04972	Pancreatic secretion
	hsa05150	*Staphylococcus aureus* infection

**Figure 5:**
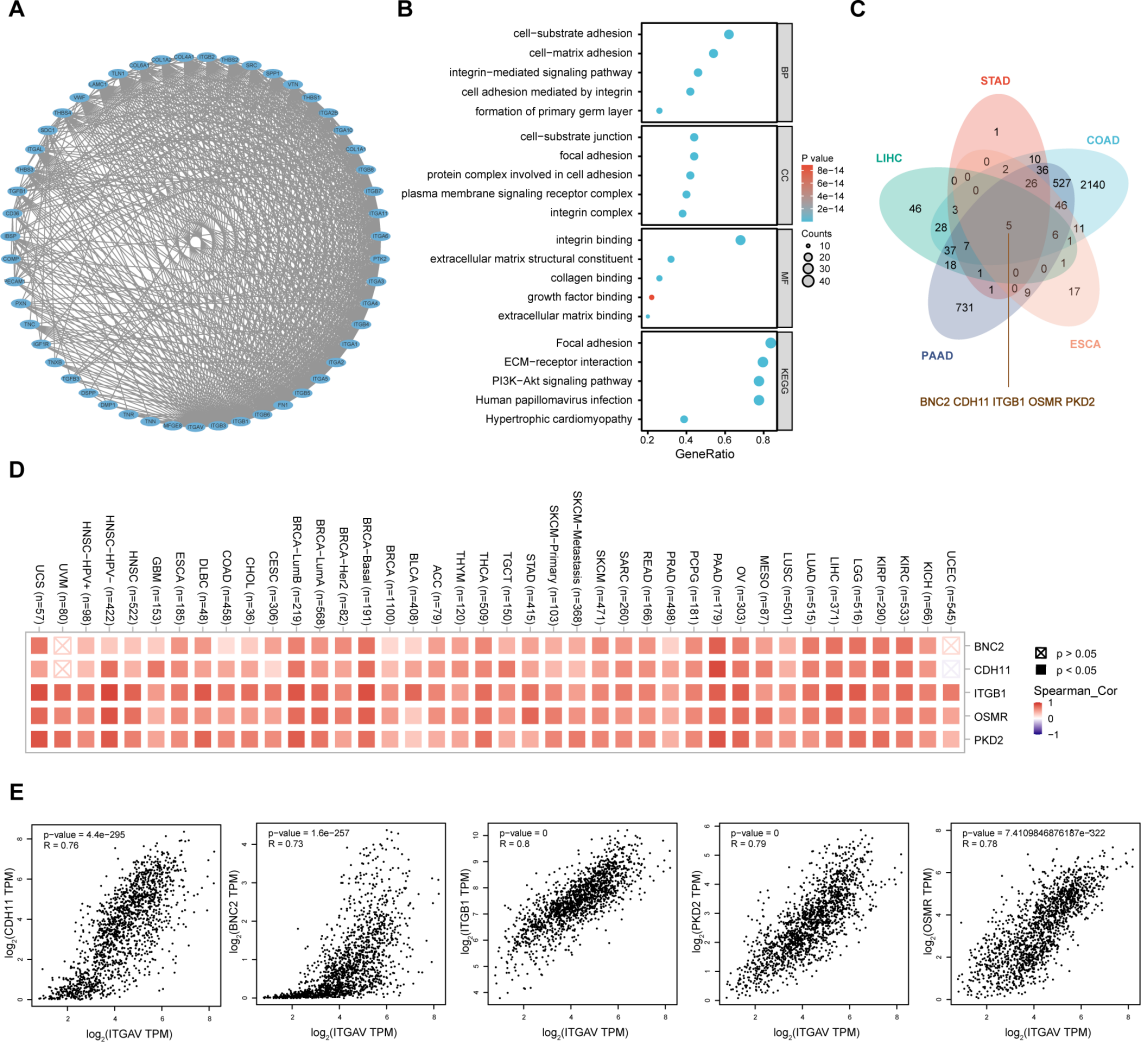
The PPI network and co-expressed genes correlation analysis of ITGAV. **(A)** The PPI network of ITGAV. **(B)** Functional enrichment analysis of the top 50 proteins associated with ITGAV. **(C)** Vein diagram showing the overlap of ITGAV co-expressed genes in digestive system cancers. **(D)** Heatmap for top 5 ITGAV-correlated genes in tumors. **(E)** expression correlation between ITGAV and genes (PKD2, CDH11, OSMR, ITGB1, and BNC2) as determined by GEPIA2.

In addition, the top 50 proteins that interact with ITGAV were extracted from the STRING database and displayed as a protein-protein interaction network (**Figure 5A**). GO/KEGG enrichment analyses were performed on these genes. The results also showed that ITGAV was closely associated with extracellular matrix remodeling and cell-substrate attachment, and KEGG pathway analysis showed that the top three enriched rankings were focal adhesion, PI3K-Akt signaling pathway, and ECM receptor interactions (**Figure 5B**), which were the main pathways regulating the migratory process of tumors.

On this basis, the cBioPortal database was used to analyze the ITGAV co-expressed genes in the above five digestive system cancers. Spearman’s correlation coefficients |R| >0.5, *P*-value <0.05 was used as the screening index. After comparing the co-expressed genes in these five digestive system cancers, five genes, PKD2, CDH11, OSMR, ITGB1, and BNC2, were further identified as being associated and interacting with ITGAV through the Venn diagram (**Figure 5C**). The heatmap created by Timer2.0 verified a significant positive correlation between these five genes and ITGAV (**Figure 5D**). Moreover, we obtained correlation analysis plots of all these five genes with ITGAV using the GEPIA2.0 database (**Figure 5E**): ITGB1 (R = 0.8), PKD2 (R = 0.79), OSMR (R = 0.78), CDH11 (R = 0.76), and BNC2 (R = 0.73). These results suggest that ITGAV plays a multifaceted and critical role in regulating digestive system cancer progression.

### 3.4 Experimentally based validation of the effect of ITGAV on the migratory movement of tumor cells

**Figure 6:**
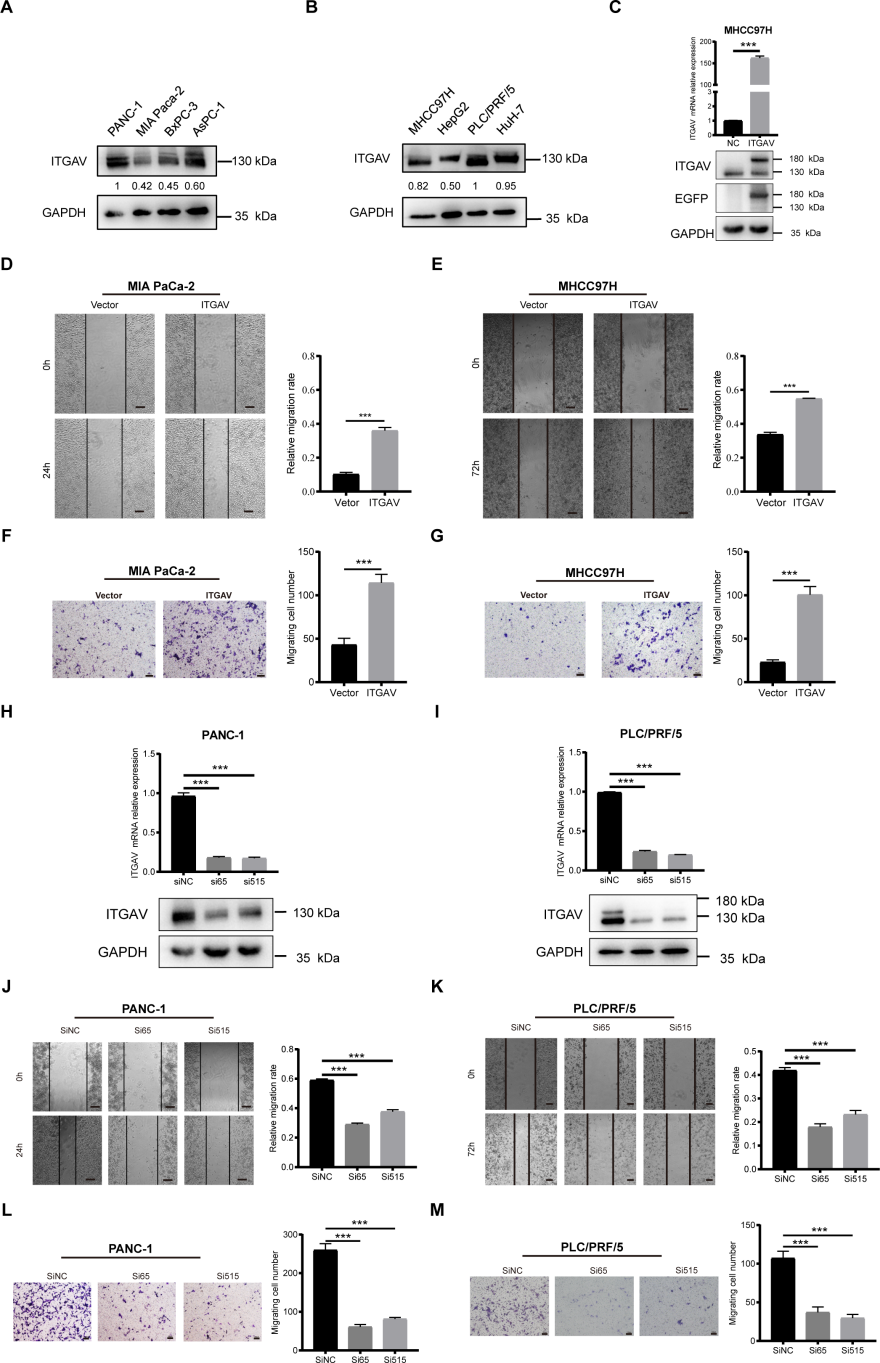
The effect of ITGAV on the migration movement of PAAD/LIHC cells. **(A, B)** Protein expression levels of ITGAV in four pancreatic cancer cell lines and four hepatocellular carcinoma cell lines. **(C)** Validation of ITGAV overexpression plasmid transfection. **(D, E)** Wound healing assay to investigate the effect of ITGAV on MIA PaCa-2 and MHCC97H migration. **(F, G)** Transwell assay were performed to investigate the effect of ITGAV on the migration of MIA PaCa-2 and MHCC97H. **(H, I)** Validation of transfection of PANC-1 and PLC/PRF/5 after knockdown of ITGAV. **(J, K)** Wound healing assay to investigate the effect of ITGAV on PANC-1 and PLC/PRF/5 migration. **(L, M)** Transwell assay were performed to investigate the effect of ITGAV on the migration of PANC-1 and PLC/PRF/5. ***P < 0.001.

Previous studies indicated that ITGAV may play a promotional role in the malignant progression of digestive system cancers by engaging in signaling pathways that enhance the migration and movement of tumor cells. To verify our findings using *in vitro* experiments, representative digestive system malignancies, specifically PAAD and LIHC, were employed to investigate the effect of ITGAV on cell migration. We first examined the endogenous expression level of ITGAV in various PAAD and LIHC cell lines and found that ITGAV was expressed at a relatively low level in MIA PaCa-2 and MHCC97H cell lines but at a high level in PANC-1 and PLC/PRF/5 cell lines (**Figures 6A, B**). Hence, ITGAV was overexpressed in the MIA PaCa-2 and MHCC97H cell lines. We constructed the ITGAV plasmid and transfected it into MHCC97H cells for validation (**Figure 6C**). The cell migration ability was detected using a wound healing assay and transwell chamber-based migration assay. For the wound healing assay, the ITGAV-overexpressed group healed more than the control group (**Figures 6D, E**). For the transwell chamber-based migration assay, the ITGAV-overexpressed group could significantly promote the cell migration number of PAAD and LIHC cells as compared to the control group after 48 h incubation (**Figures 6F, G**). Both the wound healing assay and transwell chamber-based migration assay showed that the ITGAV-overexpressed group increased the migration ability of PAAD and LIHC cells.

To investigate the results further, we interfered with ITGAV expression by transfecting cells with siRNA, which qRT-PCR and western blotting subsequently validated to confirm the reduction in ITGAV level (**Figures 6H, I**). The migration ability of PANC-1 and PLC/PRF/5 cells was reduced after knocking down ITGAV in the wound healing assay (**Figures 6J, K**). Similarly, the cell migration number of the cells was also decreased (**Figures 6L, M**). These results demonstrated that siITGAV inhibited the migration movement of PAAD and LIHC cells. Collectively, ITGAV was involved in promoting the migration and movement of PAAD and LIHC cells, thereby further enhancing the malignant progression of the tumors.

### 3.5 Correlation between ITGAV and immune infiltration in digestive system cancers

**Figure 7:**
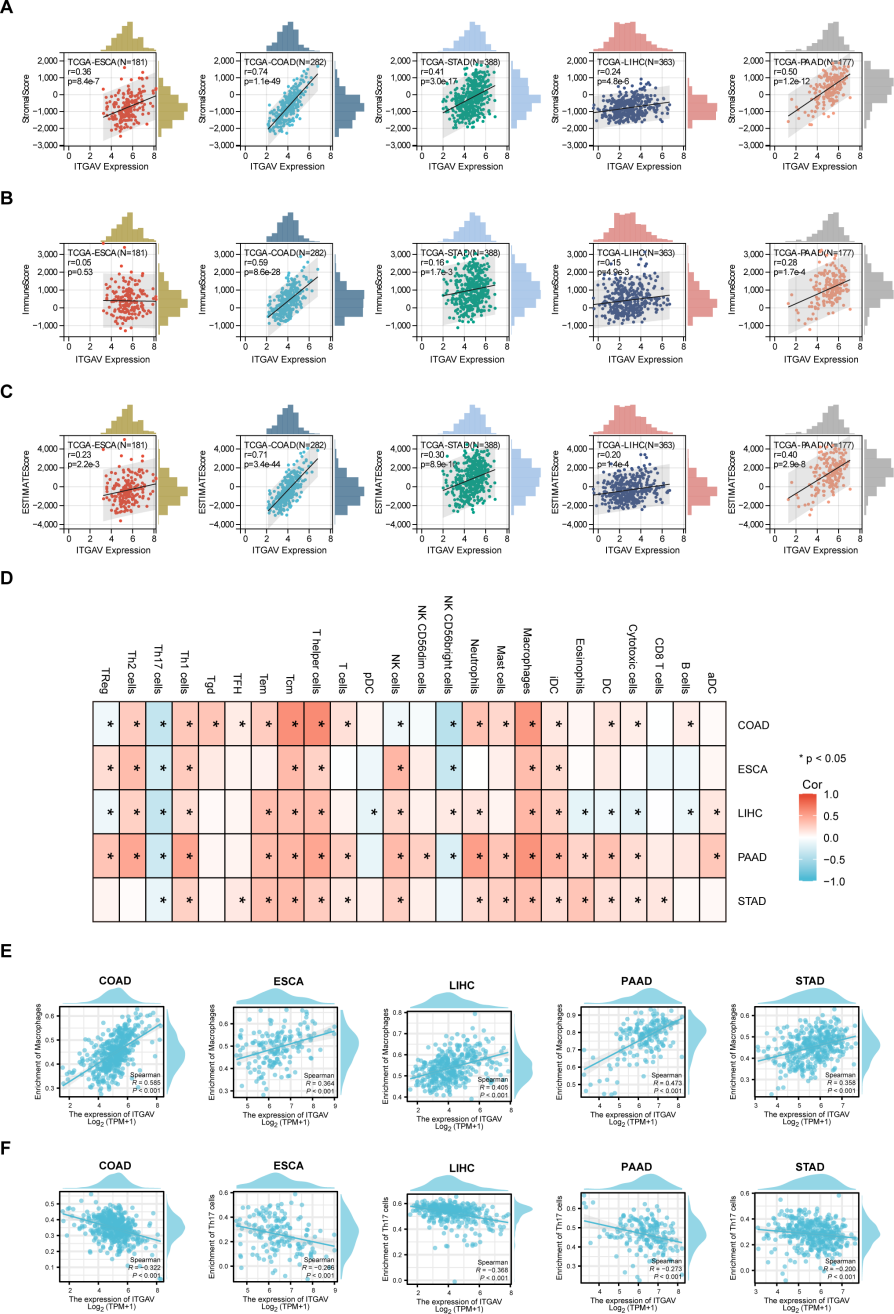
Correlation between ITGAV expression and immune cell infiltration in digestive system cancers. **(A–C)** Correlation between ITGAV expression and StromalScore, ImmuneScore, ESTIMATEScore. **(D)** ssGSEA analysis of the correlation between ITGAV expression and immune cell infiltration. *P* < 0.05 = “*” **(E, F)** Scatter plots showing the correlation between ITGAV expression and macrophage and Th17 cell infiltration in digestive system cancers.

Since the extracellular matrix can influence the infiltration function of immune cells in the tumor microenvironment (TME), which plays a part in the occurrence and development of tumors (23, 24). Then, we sought to investigate whether ITGAV is involved in immunomodulatory processes. First, we explored the correlation between ITGAV expression and the TME by ESTIMATE analysis. A significant positive correlation between ITGAV expression and StromalScore and ESTIMATEScore was observed in COAD, STAD, LIHC, and PAAD, while no significant correlation was observed for ImmuneScore in ESCA (**Figures 7A–C**). Furthermore, the notable associations observed between ITGAV and immune checkpoints, homologous recombination defects (HRD), microsatellite instability (MSI), and tumor mutation burden (TMB) indicate the involvement of ITGAV in tumor immunity, as depicted in **Supplementary Figures S3A–D**. Next, the correlation between ITGAV and 24 immune cell infiltration was further investigated using the ssGSEA algorithm. The results indicated that ITGAV was positively correlated with the infiltration levels of most immune cells, and in particular, a significant positive correlation with macrophage infiltration was found in all five digestive system cancers (**Figure 7D**). On the other hand, ITGAV showed a significant negative correlation with the infiltration level of Th17 cells among these five digestive system cancers (**Figure 7D**). As is known, Macrophages and T helper cell 17 (Th17), important and influential cell types in the tumor microenvironment, directly impact tumor development and metastasis (25, 26). In addition, correlation analysis also indicates that ITGAV may be involved in tumor immunity by regulating macrophage and Th17 cell function (**Figure 7E, F**). These results revealed a close relationship between ITGAV and immune infiltration.

The interaction between Cancer-associated fibroblasts (CAFs) and the TME is thought to be another key factor driving tumor progression (27). Therefore, we sought to determine whether ITGAV could participate in tumor immunity by modulating the function of CAFs. The results of our TIMER algorithm showed that the expression of ITGAV was positively correlated with the infiltration of CAFs in COAD, ESCA, LIHC, PAAD, and STAD (**Supplementary Figure S4A**). To make the results more convincing, we further examined the association between ITGAV and biomarkers of CAFs (ACTA2, COL1A1, FAP, VIM). The scatter plot results show that ITGAV correlates highly with CAF biomarkers (**Supplementary Figure S4B**). These results suggest that ITGAV may play a crucial role in the tumor microenvironment by affecting the function of macrophages, Th17 cells, and CAFs, which provides new targeting strategies for tumor therapy.

**Figure 8:**
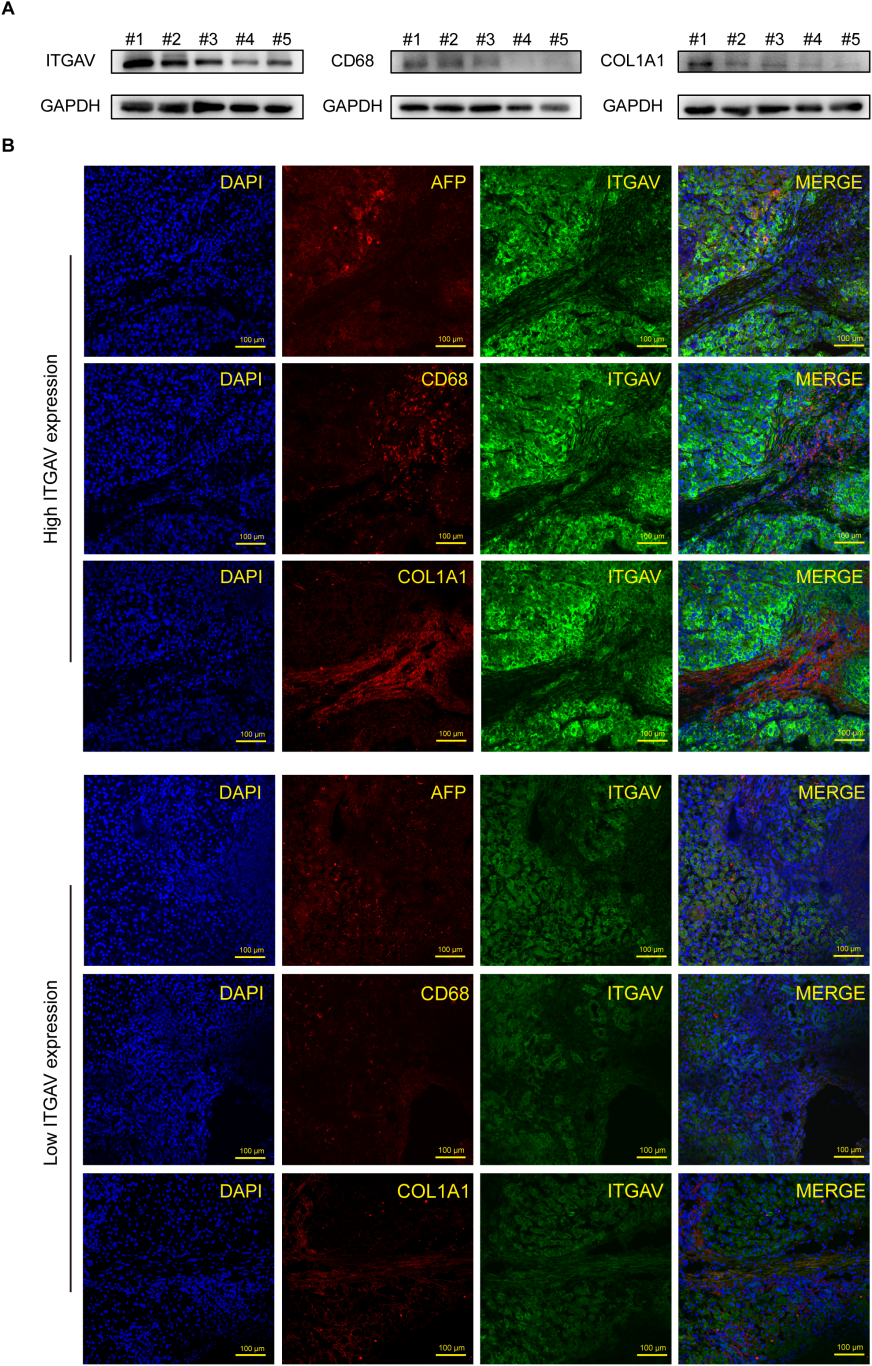
Correlation between ITGAV expression and immune cell infiltration in hepatocellular carcinoma. **(A)** Protein expression levels of ITGAV, CD68, and COL1A1 in 5 LIHC tissues. **(B)** Immunofluorescence staining of ITGAV or CD68 (green) and AFP or COL1A1 (red) in LIHC tissue from patients. Nuclear staining with DAPI. Scale bar, 100 μm.

Therefore, In order to deeply investigate the close link between ITGAV and immune infiltration, we examined the effect of ITGAV on immune infiltration of macrophages and CAFs by using LIHC tissues with different ITGAV expression levels. Firstly, we detected the expression level of ITGAV protein in clinical tissue samples from 5 patients with liver cancer, and found that the expression level of ITGAV in liver cancer tissues was positively correlated with the expression levels of CD68 (macrophage marker) and COL1A1 (CAFs marker) (**Figure 8A**). In addition, immunofluorescence results showed significant overlap between ITGAV and alpha-fetoprotein (AFP), a commonly used biomarker in LIHC tissues, which further validated previous results (**Figure 8B**). Meanwhile, we observed that the expression level of ITGAV was significantly positively correlated with the aggregation of the macrophage marker CD68 and the CAFs marker COL1A1.The LIHC tissues with high expression of ITGAV showed stronger red and green fluorescence signals than those with low expression of ITGAV, which suggested that tumor tissues from patients with high expression of ITGAV may contain more macrophages and CAFs. These results further supported the possibility that ITGAV may influence tumor progression by regulating the infiltration of macrophages and CAFs.

### 3.6 Immunogenomic analysis of ITGAV in digestive system cancers

To comprehensively assess the relationship of ITGAV with immune infiltration and immunomodulation, we analyzed the correlation between the abundance of tumor-infiltrating lymphocytes (TILs) and ITGAV methylation, MHC molecules, immunostimulators, immunoinhibitors, chemokines, and chemokine receptors by Gene Set Variation Analysis(GSVA) (**Supplementary Figure S5**). Heat map results showed that ITGAV methylation was positively correlated with most immune cell infiltration in ESCA and STAD, whereas it was negatively correlated in COAD and PAAD (**Supplementary Figure S5A**). Moreover, in LIHC, PAAD, and COAD, ITGAV expression was positively correlated with most MHC molecules (**Supplementary Figure S5B**). Notably, ITGAV was positively associated with most immunostimulators and immunoinhibitors in five digestive system cancers: LIHC, COAD, STAD, ESCA, and PAAD (**Supplementary Figures S5C–D**). Additionally, most cytokines and their receptors were also positively associated with ITGAV (**Supplementary Figures S5E–F**).

**Figure 9:**
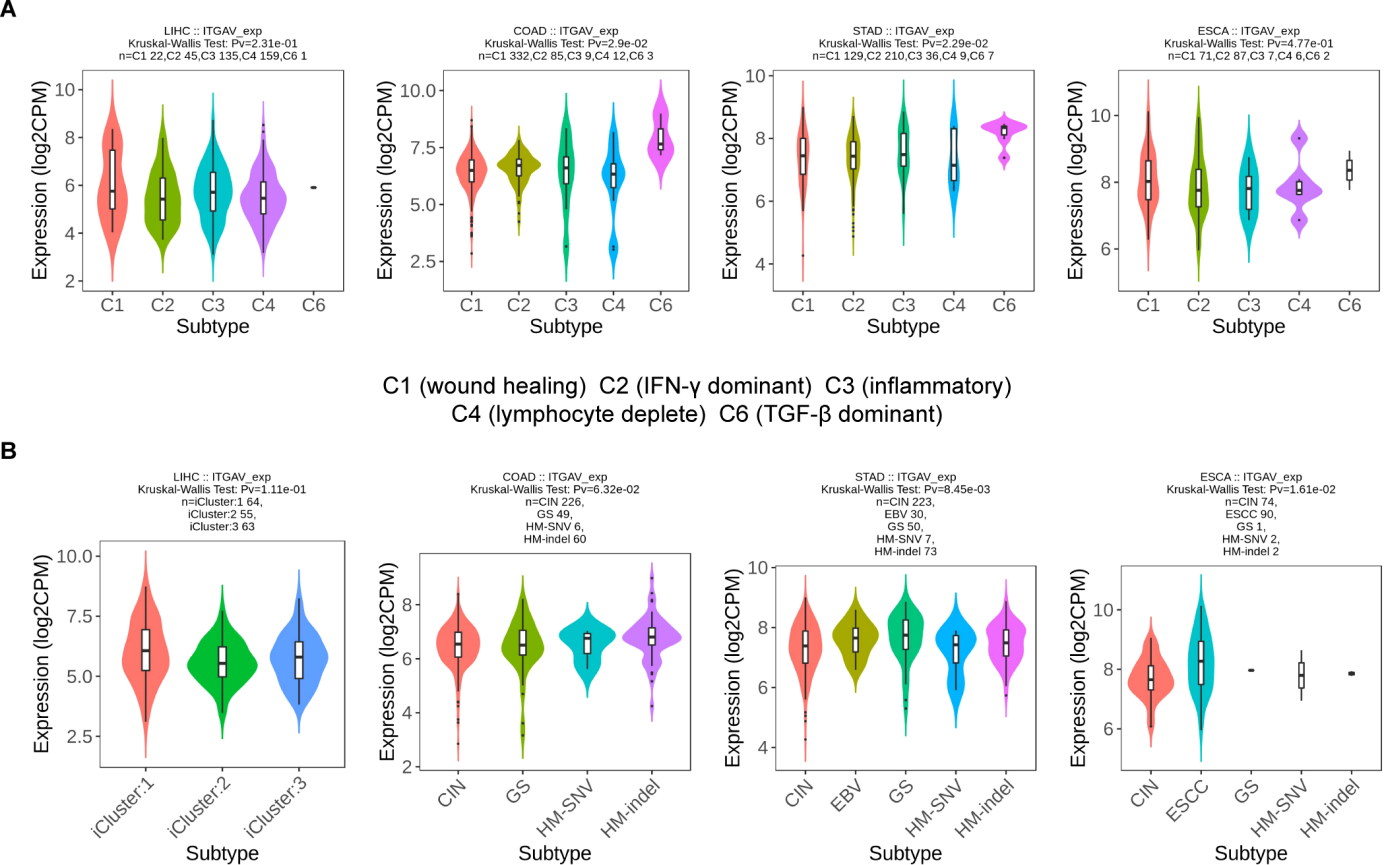
ITGAV expression in immune and molecular subtypes of digestive system cancers. **(A)** The relationship between ITGAV expression and immune subtypes. **(B)** The relationship between ITAGV expression and molecular subtypes.

Based on our previous findings, we identified that the expression level of ITGAV influences OS in numerous digestive system cancers (**Figure 2**). Hence, we proceeded to analyze the expression patterns of ITGAV in immune subtypes and molecular subtypes of these cancers using the TISIDB database. The results showed that ITGAV expression in STAD (5 subtypes) and COAD (5 subtypes) correlated with their immune subtypes (**Figure 9A**). ITGAV expression in ESCA (5 subtypes) and STAD (5 subtypes) was associated with its molecular subtype (**Figure 9B**). These findings provide significant insights into the role of ITGAV in tumor immunomodulation and provide a foundational basis for developing immunotherapeutic strategies targeting ITGAV in the clinic.

## 4 Discussion

Digestive system cancers are widely considered to be one of the most common causes of cancer death worldwide including COAD, LIHC, ESCA, PAAD, and STAD, which were characterized by a correspondingly high morbidity and mortality rate (28–30). Although there are many studies about early diagnosis and immune-based intervention strategies in digestive system cancers, they have not been well applied in clinical therapy and remain some challenges. Consequently, it is crucial to explore novel, effective, and specific biomarkers and immunotherapy targets to achieve early detection, treatment, and prognostic evaluation of cancer.

Integrins were crucial cell surface receptors that were involved in mediating cell adhesion and signaling transduction which played an important role in regulating cell motility, survival, proliferation differentiation, and so on (31–33). ITGAV, a member of the integrin receptor family that mediates the attachment of cells to the extracellular matrix, has been associated with cell structure and function (34). Importantly, previous results have shown that ITGAV has a significantly high expression in tumors such as HNSCC, and PCa in recent years (13, 17). In addition, significantly high expression of ITGAV had also been observed in highly prevalent digestive system cancers such as gastric cancer (35). However, systematic studies and analyses of ITGAV expression in the broad category of digestive system cancers were still inadequate. In our initial analysis of tumor samples and corresponding normal samples from multiple databases, we observed that ITGAV mRNA and protein expression were consistently elevated in various digestive system cancers represented by LIHC, PAAD, COAD, ESCA, and STAD. Furthermore, we observed a consistent increase in ITGAV expression level that correlated with advancing tumor malignancy across various categories, including T-stage, N-stage, M-stage, and pathological stage. These findings suggested that we should pay attention to the relationship between ITGAV expression and digestive system cancers, which may contribute to tumorigenesis and development.

Tumor biomarkers are of great significance for the evaluation of prognosis, the early diagnosis of tumors, and the formulation of personalized treatment plans (36, 37). It had been demonstrated that ITGAV was a prognostic biomarker of relapse in cutaneous squamous cell carcinomas (cSCCs) that would allow improved patient stratification (38). It has also been reported in the literature that in ESCA, ITGAV expression was associated with a shorter overall survival in the group of patients who underwent primary surgery, but not in the group of patients who received neoadjuvant therapy prior to surgery, suggesting that ITGAV could be a prognostic factor in ESCA (39). Moreover, In a recent study by Liu et al, cilengitide, a specific antagonist of ITGAV, was found to significantly inhibit LIHC invasion and metastasis in combination with the selective gamma-secretase inhibitor LY3039478, improving the prognosis not only of patients with LIHC but also of patients with other advanced solid tumors (12). It prompted us to explore whether ITGAV can be used as an effective and reliable biomarker for diagnosis or prognosis. For this reason, our study started with a focus on the relationship between ITGAV gene expression and OS, DSS, PFI, and diagnostic accuracy in digestive system cancers. Our findings showed that ITGAV upregulation was positively correlated to the poor prognosis and has high diagnostic accuracy (AUC > 0.7), suggesting that it could be a promising potential prognostic and diagnosis biomarker for digestive system cancers. In summary, ITGAV demonstrated significantly elevated expression levels in digestive system cancers, which were closely associated with poor survival outcomes and exhibited good diagnostic value, indicating a significant connection between ITGAV and the malignant progression of the tumors.

ITGAV is thought to affect multiple aspects of tumor proliferation, survival, invasion, metastasis, immunosuppression, and drug resistance. Previous research has demonstrated that ITGAV is a crucial gene involved in regulating cellular migration, proliferation, and metastasis. Baghmisheh et al. demonstrated that ITGAV can promote lung cancer migration, invasion, and metastasis by mediating the expression of CASZ1 (40). In the research of Wang et al., high expression of ITGAV was associated with poorer prognostic outcomes in gastric cancer, and the low expression of ITGAV resulted in inhibition of proliferation, migration, and invasion in GC cells (41). However, few previous studies focused on the role of ITGAV in digestive system cancers, and to fill this gap, we conducted a large number of data analyses in digestive system cancers. Through GO and KEGG analysis of the DEGs of ITGAV in digestive system cancers, we found that ITGAV was closely related to the organization processes of the extracellular matrix and was enriched in pathways regulating tumor cell migration and motility. Subsequently, the PPI network of the top 50 ITGAV-related proteins was constructed, and functional enrichment analysis of these genes showed that ITGAV was closely related to tumor cell migration to regulate the progression of digestive system cancers, such as focal adhesion, PI3K-Akt signaling pathway, and ECM receptor interactions. Afterward, to definite the functional role of ITGAV in tumor metastasis mechanisms, we further validated the above results *in vitro*. We utilized the wound healing assay and transwell chamber-based migration assay after validating the successful construction of the cell models of PAAD and LIHC at the mRNA and protein levels. The experimental results demonstrated that the migration ability of the cell lines with the overexpression of ITGAV was significantly enhanced, whereas the knockdown of ITGAV significantly suppressed cell migration and motility, indicating the pivotal role of ITGAV in promoting the cancer cell migration movement in PAAD and LIHC. Based on these results, we found that ITGAV plays a crucial role in the structure and function of the digestive tract, potentially promoting the progression of digestive system cancers by influencing cellular migratory capacity, but more *in vivo* and *in vitro* studies are needed in the future to elucidate deeper mechanisms.

The tumor microenvironment (TME), comprising diverse immune cell types, plays a significant role in tumor progression, metastasis, and immunotherapy (41). Tumor-infiltrating immune cells markedly influence both the TME and tumor behavior (42, 43). For example, ITGAV was an immunomodulatory protein that promoted T-cell activation during priming to enhance anticancer efficacy in tumor models (44). Also reported in the literature, ITGAV can promote the progression of cholangiocarcinoma (CCA) by affecting the infiltration of neutrophils (45). However, more studies are needed for further exploration of the connection between ITGAV and TME. We discovered that ITGAV expression was positively related to ESTIMATEScore across different digestive system cancers and was associated with the infiltration level of most immune cells, such as macrophages, Th17 cells, etc. Many researchers have confirmed that macrophages were associated with tumor development and poor clinical prognosis (46–48). Interestingly, Th17 cells could promote the anti-tumor immune response by recruiting and activating B and T cells and were significantly correlated with improved outcomes in HNSCC patients (49). In our study, it was noteworthy that ITGAV was positively correlated with macrophages indicating that ITGAV may be involved in the activation and regulation of macrophages to promote recruitment. Conversely, ITGAV showed a negative correlation with Th17 cells suggesting that it may be related to pathways inhibiting the infiltration of Th17 cells, thereby fostering an environment conducive to tumor growth. These results suggested that ITGAV may be involved in tumor immunology regulation by influencing macrophages and Th17 functions. Furthermore, the expression of ITGAV was closely associated with various immune checkpoints, TMB, MSI, and HRD, as well as some immune and molecular subtypes in different types of digestive system cancers. In general, ITGAV displayed a close linkage with immune regulation to provide valuable theoretical proof that can be used as a new immune-related therapeutic target in digestive system cancers.

Additionally, CAFs also were crucial components of the TME, which can promote cancer cell progression and immune evasion (50). Previous studies showed that CAFs can contribute to promoting prostate cancer stem cell (PCSC) growth and survival by releasing signaling molecules and modifying the surrounding environment (51). The findings of our study showed that the expression of ITGAV exhibited a significant positive relationship with the infiltration of CAFs in digestive system cancers. Meanwhile, we observed that ITGAV expression was positively correlated with many CAFs characteristic markers, such as ACTA2, COL1A1, FAP, and VIM. It suggested that ITGAV may contribute to creating a conducive environment for tumor growth, dissemination, and metastasis by regulating the infiltration of CAFs, thereby informing the development of clinically effective new treatments for digestive system cancers.

However, more experiments are still needed to reveal the mechanism of action of ITGAV in digestive system cancers. Therefore, we still need to do subsequent animal experiments. Additionally, the combination of targeted ITGAV and antitumor drugs or immune checkpoint inhibitors enhances the efficacy of antitumor immunotherapy and seeks more effective clinical treatment strategies.

In conclusion, our study elucidated the role of ITGAV in digestive system cancers from various aspects. It was discovered that ITGAV regulates the migration and movement of digestive system cancer cells through bioinformatics and experimental approaches. Secondly, ITGAV is associated with pathways for the construction and regulation of the TME, particularly in the infiltration of immune cells and CAFs. These findings contribute to a comprehensive understanding of the functions of ITGAV in prognostic and immunotherapy in digestive system cancers.

## Data Availability

The original contributions presented in the study are included in the article/**Supplementary Material**. Further inquiries can be directed to the corresponding authors.
