# Supplementary material for: Comprehensive analysis of the multifaceted role of ITGAV in digestive system cancer progression and immune infiltration
Source: Front Immunol. 2025 Feb 13;16:1480771. doi: 10.3389/fimmu.2025.1480771 (PMC11864929; doi:10.3389/fimmu.2025.1480771)
Supplement: Supplementary file 1 [file DataSheet1.zip › Supplementary_Material.docx]

Supplementary Material

# Supplementary Figures

**Supplementary Figure 1.** Analysis of ITGAV expression in the GEPIA database. **(A)** ESCA. **(B)** PAAD. **(C)** STAD. **(D)** LIHC. **(E)** COAD.

**Supplementary Figure 2.** ITGAV mRNA expression levels in tumor tissue samples from patients with gastrointestinal tumors of different degrees of malignant evolution. **(A)** Pathologic N stage. **(B)** Pathologic T stage. **(C)** Pathologic M stage. **(D)** Pathologic stage. **P* < 0.05, ***P* < 0.01, ****P* < 0.001. ns, not statistically significant.

**Supplementary Figure 3.** Correlations of ITGAV expression with immune checkpoints, TMB, MSI, and HDR. **(A)** immune checkpoints. **(B)** TMB. **(C)** MSI. **(D)** HDR.

**Supplementary Figure 4.** Correlation between ITGAV and CAF infiltration in gastrointestinal tumors. **(A)** The scatter plot shows the correlation between ITGAV expression and CAF infiltration. **(B)** The scatter plot shows the correlation between ITGAV expression and CAF markers ACTA2/COL1A1/FAP/VIM.

**Supplementary Figure 5.** Correlation of ITGAV with TILs and immunoregulation-related genes in gastrointestinal tumors. **(A)** Correlations between ITGAV methylation and TILs. **(B)** Correlations between ITGAV expression and MHC molecules. **(C)** Correlations between ITGAV expression and immunoinhibitors. **(D)** Correlations between ITGAV expression and immunostimulators. **(E)** Correlations between ITGAV expression and chemokines. **(F)** Correlations between ITGAV expression and chemokine receptors.
