# Supplementary figures and images for: Comprehensive analysis of the multifaceted role of ITGAV in digestive system cancer progression and immune infiltration
Source: Front Immunol. 2025 Feb 13;16:1480771. doi: 10.3389/fimmu.2025.1480771 (PMC11864929; doi:10.3389/fimmu.2025.1480771)

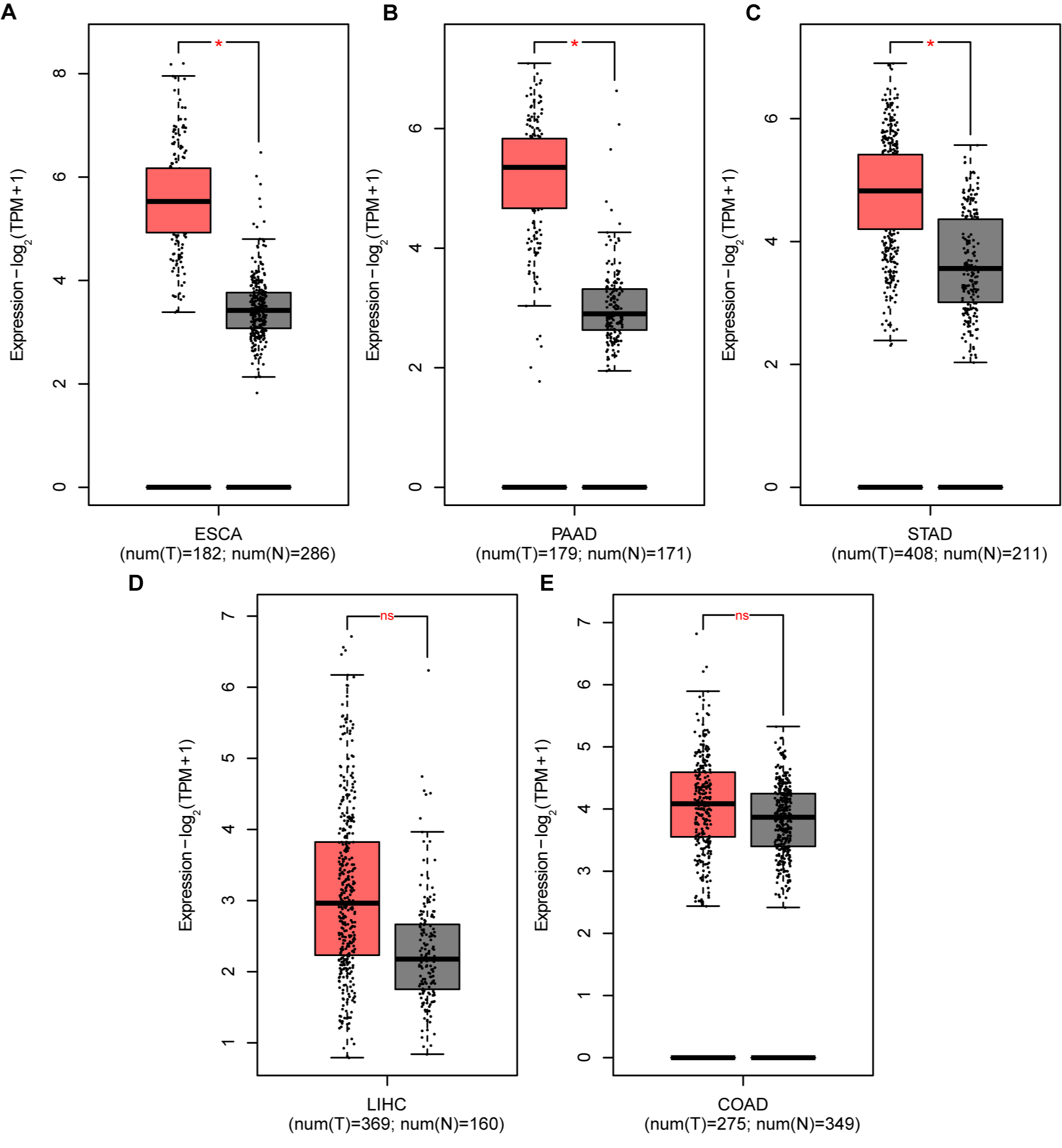

Supplement: Supplementary file 1 [file DataSheet1.zip › Supplementary Figure 1.tif]

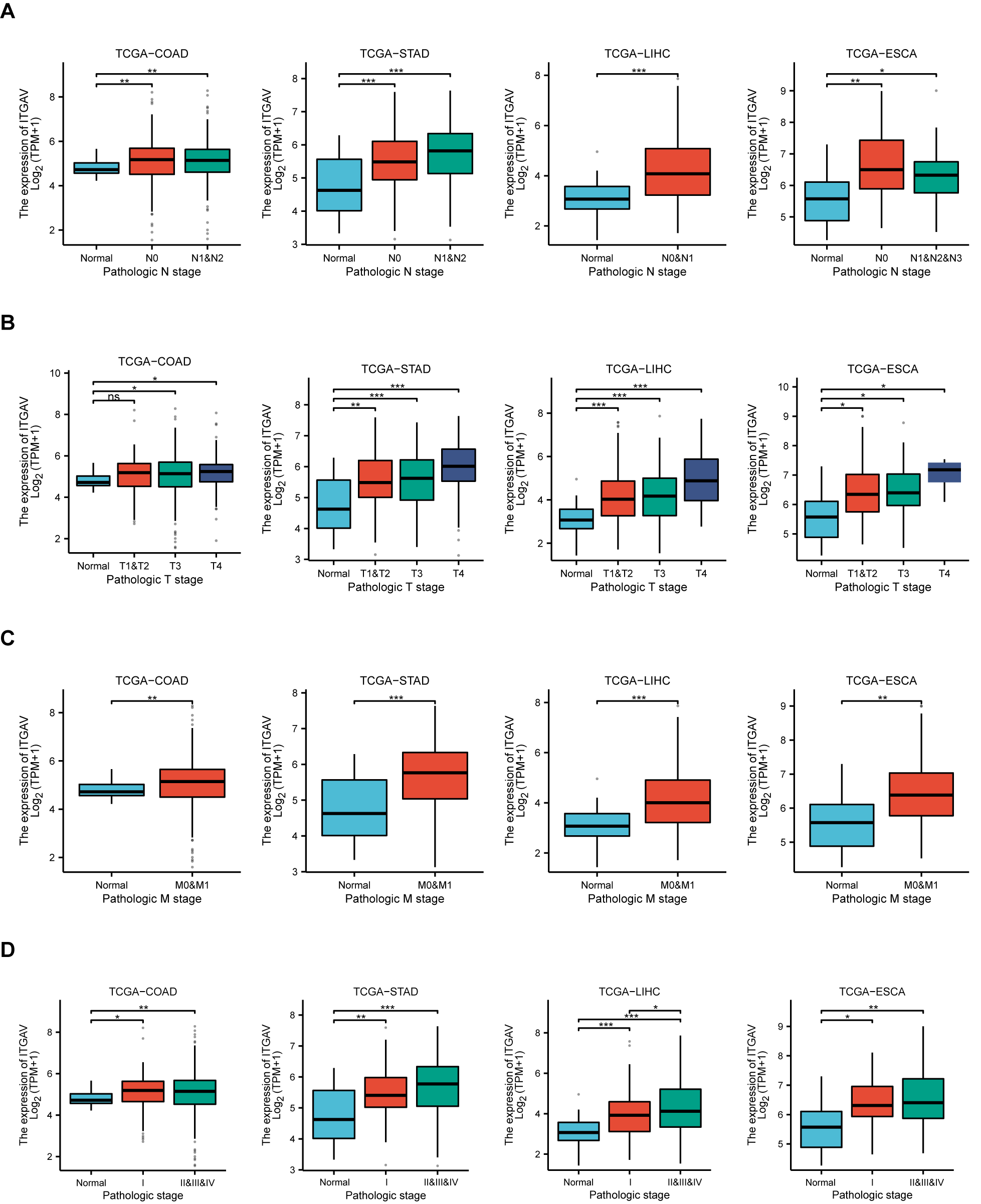

Supplement: Supplementary file 1 [file DataSheet1.zip › Supplementary Figure 2.tif]

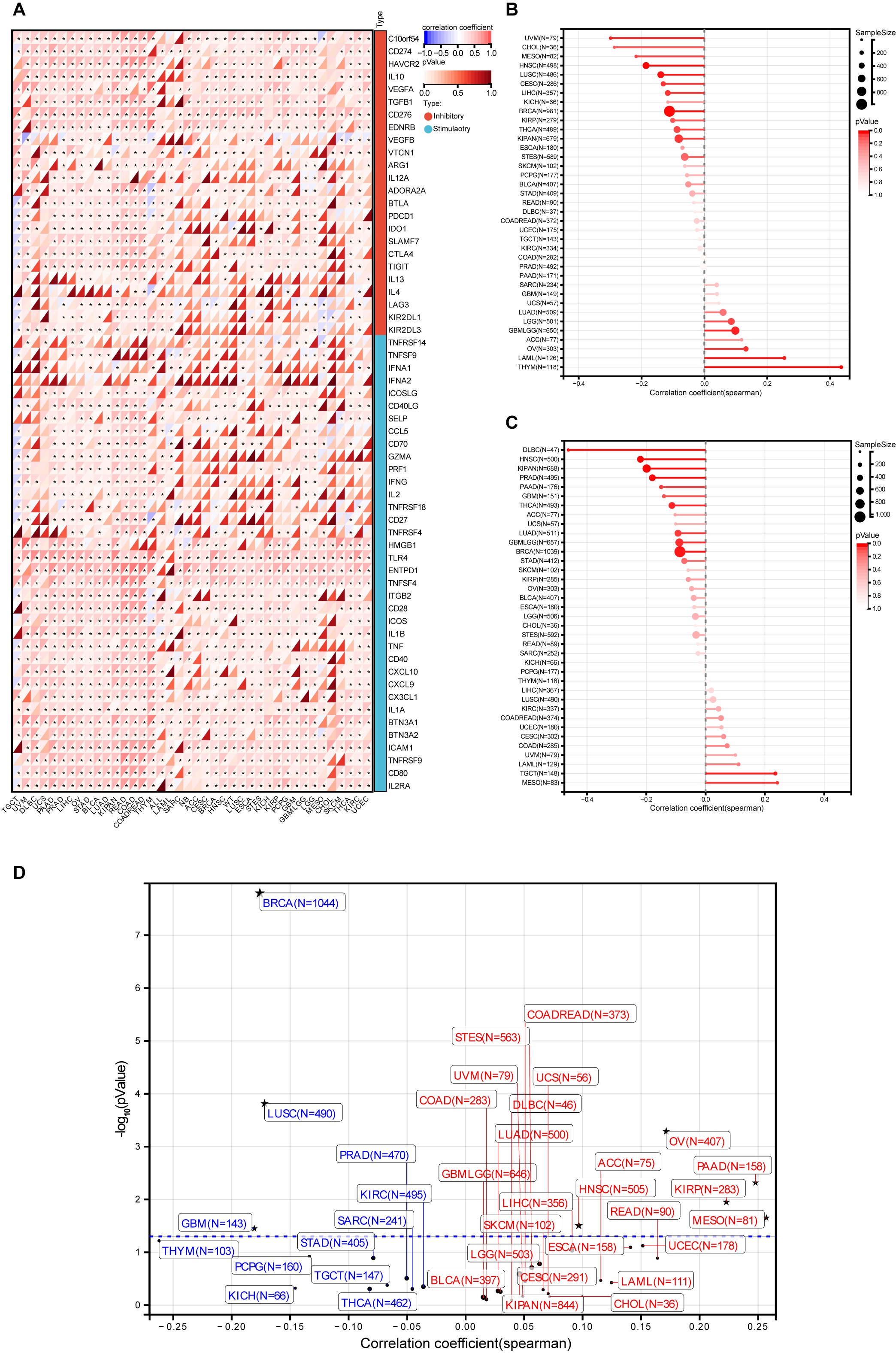

Supplement: Supplementary file 1 [file DataSheet1.zip › Supplementary Figure 3.tif]

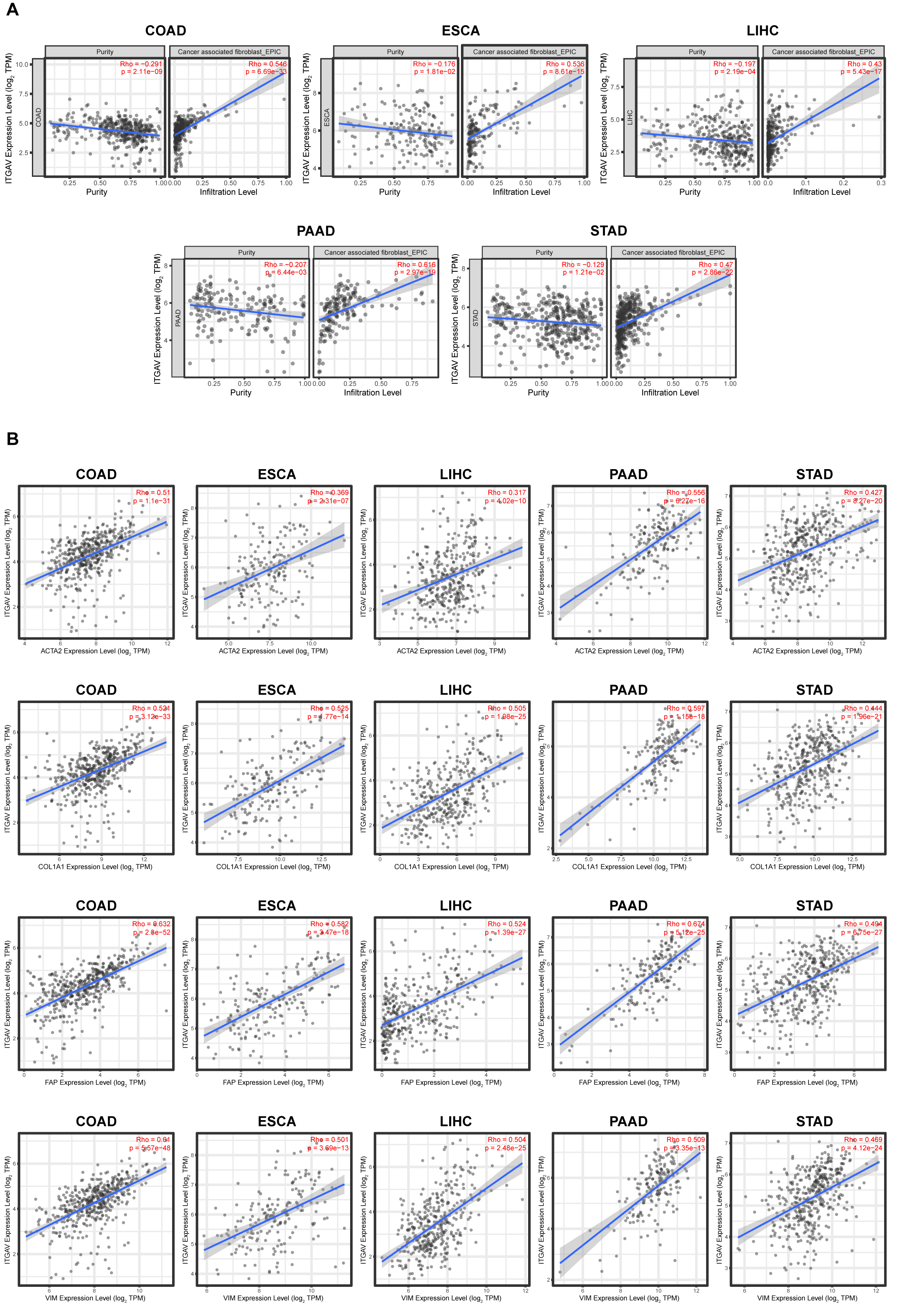

Supplement: Supplementary file 1 [file DataSheet1.zip › Supplementary Figure 4.tif]

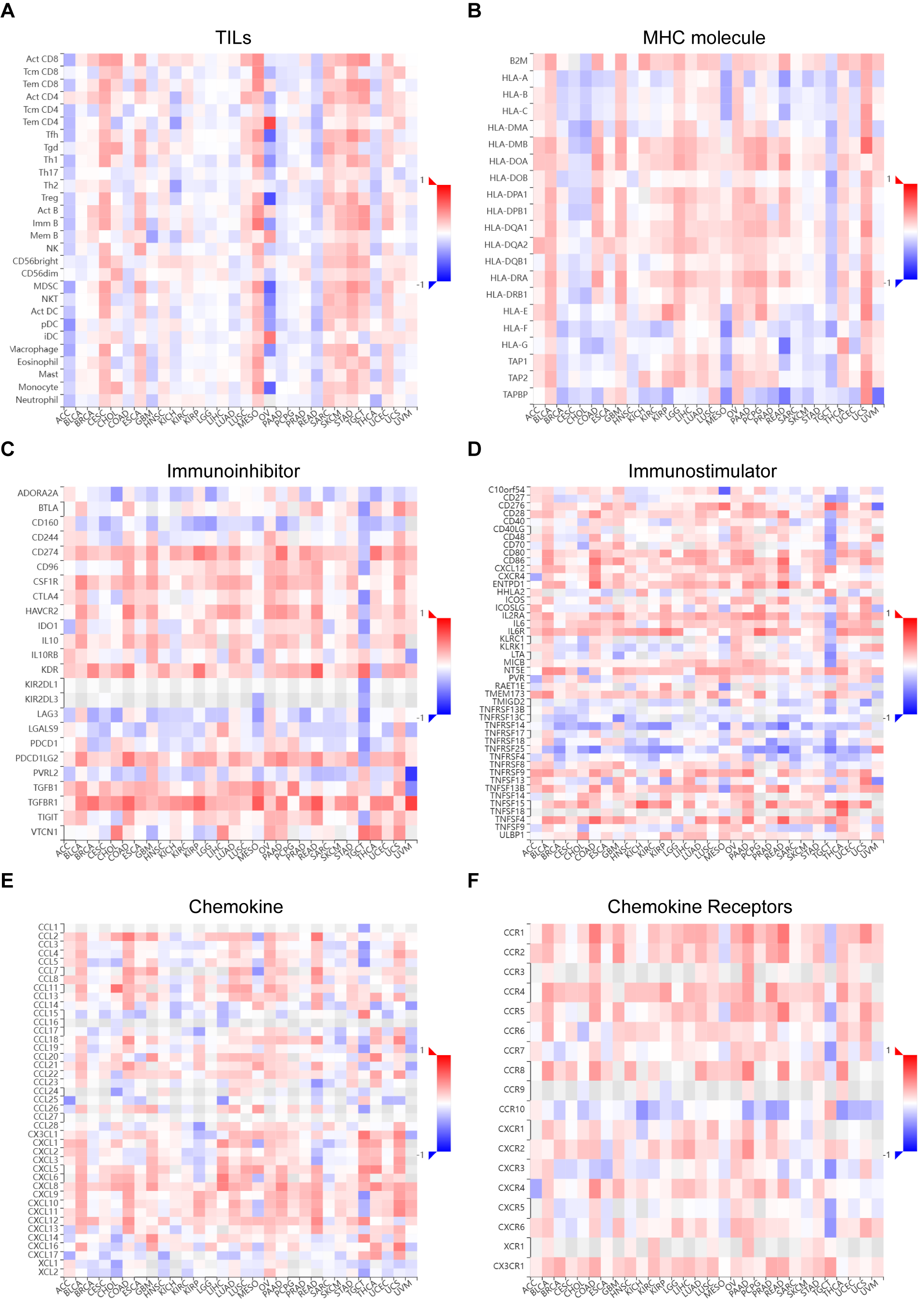

Supplement: Supplementary file 1 [file DataSheet1.zip › Supplementary Figure 5.tif]
